# 2020–2024年广东地区血液内科血液标本分离菌流行病学变迁分析

**DOI:** 10.3760/cma.j.cn121090-20250328-00154

**Published:** 2025-06

**Authors:** 烨欣 林, 希铭 陈, 岩 张, 窘 王, 雯雯 梁, 勤虹 谢, 华亮 陈, 秋雪 邓, 旭 杨, 宁静 刘, 译靖 王, 明欣 李, 杨进 陈, 雅婷 赵, 南豪 何, 嘉康 陈, 书念 肖, 超 卓

**Affiliations:** 1 广州医科大学附属第一医院（广东省耐药菌监测和质量控制中心），广州呼吸健康研究院，广州 510030 First Affiliated Hospital of Guangzhou Medical University, Guangzhou Institute of Respiratory Health, Guangzhou 510030, China; 2 广州医科大学呼吸疾病全国重点实验室，广州 510030 Guangzhou Medical University, State Key Laboratory of Respiratory Disease, Guangzhou 510030, China

**Keywords:** 细菌耐药监测, 血液内科, 血流感染, 碳青霉烯耐药革兰阴性菌, 新抗菌药物, Antimicrobial resistance surveillance, Hematology department, Bloodstream infections, Carbapenem-resistant Gram-negative organisms, Novel antimicrobial agents

## Abstract

**目的:**

探讨2020–2024年广东省56家成员单位血液内科血液标本病原菌分布和常见抗菌药物耐药率和敏感率变迁，比较性明晰血液内科血流感染的流行病学特征。

**方法:**

对2020–2024年广东省56家成员单位血液内科、呼吸内科和重症监护病房（ICU）血液标本的临床分离株分布构成比进行分析，并对5年内检出率高的病原菌进行耐药性变化分析。分层抽取碳青霉烯耐药革兰阴性菌（CRO）进行碳青霉烯酶型检测，并检测新型抗菌药物体外药敏试验。

**结果:**

5年间，广东省56家成员单位血液内科、呼吸内科和ICU血液标本分离菌株分别为8 968、6 440和25 511株，三个科室病原菌分布构成比差异有统计学意义（*P*<0.001）。其中，血液内科占比高的细菌分别是大肠埃希菌（24.1％）、肺炎克雷伯菌（17.5％）、铜绿假单胞菌（11.7％）、凝固酶阴性葡萄球菌（15.2％）和金黄色葡萄球菌（5.1％）。耐药分析发现，大肠埃希菌和肺炎克雷伯菌对美罗培南耐药率分别从2020年6.7％和5.8％上升至2024年14.0％和15.8％；铜绿假单胞菌对美罗培南和亚胺培南耐药率从2020年6.2％和10.2％下降至2024年1.9％和6.1％；而鲍曼不动杆菌对常见抗菌药物耐药变迁呈现先下降再上升的趋势。金黄色葡萄球菌对常见抗菌药物敏感率整体比凝固酶阴性葡萄球菌高，无糖肽类和利奈唑胺耐药株。屎肠球菌对万古霉素耐药率从2020年0上升至2024年23.1％。主动监测CRO菌株的碳青霉烯酶表型，80％大肠埃希菌携带*bla*NDM、90％肺炎克雷伯菌携带*bla*KPC、10％铜绿假单胞菌携带*bla*VIM和100％鲍曼不动杆菌携带*bla*OXA-23。对针对CRO的药物敏感性分析显示，碳青霉烯耐药大肠埃希菌（CRECO）对替加环素、多黏菌素和氨曲南/阿维巴坦耐药率为0；碳青霉烯耐药肺炎克雷伯菌（CRKPN）对氨曲南/阿维巴坦、头孢他啶/阿维巴坦和亚胺培南/瑞莱巴坦的耐药率为0；碳青霉烯耐药铜绿假单胞菌（CRPAE）对阿米卡星和多黏菌素B敏感率达95.0％，对头孢他啶/阿维巴坦耐药率为45.0％；碳青霉烯耐药鲍曼不动杆菌（CRABA）对舒巴坦/杜洛巴坦敏感率达100.0％，90％最低抑菌浓度（MIC90）为2 µg/ml，对依拉环素50％最低抑菌浓度（MIC50）和MIC90分别为1 µg/ml和2 µg/ml。

**结论:**

血液内科血液标本分离菌构成与呼吸内科和ICU比较有显著差异。CRO检出率呈升高趋势，但CRO对新型抗菌药保持高度敏感性。

## Abstract (en)

**Objective:**

To investigate the pathogen distribution, temporal trends in the rates of antimicrobial resistance, and susceptibility of bloodstream isolates and comparatively explore the epidemiological characteristics of bloodstream infections in hematology departments across 56 healthcare facilities in Guangdong Province from 2020 to 2024.

**Methods:**

A multicenter analysis was conducted to evaluate the constituent ratio of different pathogens isolated from clinical isolate data from bloodstream specimens in hematology, respiratory, and intensive care unit（ICU）departments across 56 healthcare facilities in Guangdong Province（2020–2024）, and antimicrobial resistance trends in pathogens with high-detection rate over 5 years were assessed. Carbapenem-resistant Gram-negative organisms（CRO）were randomly sampled for carbapenemase gene detection and in vitro antimicrobial susceptibility tests with novel antimicrobial agents.

**Results:**

From 2020 to 2024, a total of 8 968, 6 440, and 25 511 bloodstream isolates were identified in the hematology, respiratory, and ICU departments, respectively, across 56 participating facilities in Guangdong Province, with significant differences in the pathogen constituent ratio among departments（*P*<0.001）. Notably, the hematology department demonstrated a predominance of *Escherichia coli*（24.1％）, *Klebsiella pneumoniae*（17.5％）, *Pseudomonas aeruginosa*（11.7％）, *coagulase-negative Staphylococci*（15.2％）, and *Staphylococcus aureus*（5.1％）. In the resistance analysis, the rates of meropenem resistance of *Escherichia coli* and *Klebsiella pneumonia* increased from 6.7％ and 5.8％（2020）to 14.0％ and 15.8％（2024）, respectively. Conversely, *Pseudomonas aeruginosa* exhibited a declining trend in the rate of meropenem resistance（6.2％ to 1.9％）and imipenem（10.2％ to 6.1％）during the same period. *Acinetobacter baumannii* demonstrated a biphasic resistance pattern to common antimicrobial agents, characterized by an initial decline, followed by a rebound. In this study, the susceptibility rates to conventional antimicrobial agents were significantly higher in *Staphylococcus aureus* versus *coagulase-negative Staphylococci*, with no glycopeptide- or linezolid-resistant strains detected. Notably, the prevalence of vancomycin-resistant *Enterococcus faecium* increased from 0 in 2020 to 23.1％ in 2024. CRO carbapenemase phenotypes through active surveillance revealed that 80％ *Escherichia coli* isolates were carrying *bla*NDM, 90％ *Klebsiella pneumoniae* isolates were carrying *bla*KPC, 10％ *Pseudomonas aeruginosa* isolates were carrying *bla*VIM, and 100％ *Acinetobacter baumannii* were carrying *bla*OXA-23. The results of the antimicrobial susceptibility test in CRO revealed that carbapenem-resistant *Escherichia coli*（CRECO）demonstrated a 0 resistance rate to tigecycline, polymyxin B, and aztreonam/avibactam, whereas carbapenem-resistant *Klebsiella pneumoniae* exhibited a 0 resistance rate to aztreonam/avibactam, ceftazidime/avibactam, and imipenem/relebactam. Carbapenem-resistant *Pseudomonas aeruginosa* exhibited a 95.0％ susceptibility rate to amikacin and polymyxin B, with a 45.0％ resistance rate to ceftazidime/avibactam. In contrast, carbapenem-resistant *Acinetobacter baumannii* demonstrated complete susceptibility（100.0％）to sulbactam/durlobactam（MIC90＝2 µg/ml）, whereas eravacycline showed MIC50 and MIC90 values of 1 and 2 µg/ml, respectively.

**Conclusion:**

The pathogen constituent ratio of bloodstream isolates differed significantly among hematology, respiratory, and ICU departments. Notably, although CRO exhibited an escalating prevalence, it sustained high susceptibility to novel antimicrobial agents.

血流感染是指病原体（细菌、病毒、真菌等）侵入血液循环，并在血液中生长繁殖、产生毒素或其他代谢产物，从而引起一系列全身性感染的病理过程[1]。血液内科患者多患有严重的血液系统疾病，尤其存在化疗和造血干细胞移植等耐药菌感染高危因素，血流感染风险增加[2]。研究发现，血液系统恶性肿瘤和造血干细胞移植患者血流感染的死亡率分别为17.5％和17.6％[3]。本研究基于全国细菌耐药监测网（CARSS）平台统计数据，分析2020–2024年广东省56家成员单位血液内科血液标本细菌分布、常见抗菌药物耐药率和敏感率变迁，并与呼吸内科、重症监护病房（ICU）血液标本分离病原菌构成比进行对比分析，了解血液内科临床微生物流行病学特征；旨在为临床病原菌治疗提供准确的实验室依据。

## 材料与方法

1. 菌株来源：基于CARSS平台统计信息，收集2020年1月1日至2024年12月31日广东省56家成员单位血液内科、呼吸内科和ICU血液标本数据，合并在综合科室（如大内科、肿瘤科等）的血液内科、呼吸内科不纳入分析。

2. 药敏试验：常规药敏试验按照《全国细菌耐药监测网技术方案（2024版）》方案执行。本实验室分层抽取碳青霉烯类耐药革兰阴性杆菌（CRO）采用微量肉汤稀释法进行药敏试验。质控菌株为大肠埃希菌ATCC 25922、铜绿假单胞菌ATCC 27853、鲍曼不动杆菌ATCC 13304以及金黄色葡萄球菌ATCC 29213。

3. 判读标准：参照美国临床和实验室标准协会（CLSI）2024年M100文件的判断标准[4]，替加环素按美国食品和药品监督管理局（FDA）推荐的判断标准[5]，头孢哌酮/舒巴坦参考《CHINET中国细菌耐药监测网技术方案（2022年更新版）》进行判读。依拉环素折点参照华人抗菌药物敏感性试验委员会（CHICAST）制定的ECOFF折点进行判读[6]。

4. 特殊耐药菌株定义：CRO定义为对亚胺培南、美罗培南或厄他培南中任一种耐药者[7]。

5. 耐药基因型：利用PCR检测碳青霉烯酶中β-内酰胺酶类相关耐药基因，见表1。反应体系：DNA模板1 µl，上下游引物各1 µl，Taq酶12.5 µl，加ddH_2_O 9.5 µl至25 ml。反应条件：94 °C预变性5 min，94 °C变性45 s，55 °C退火45 s，72 °C延伸1 min，30个循环后72 °C延伸10 min[8]–[9]。

**表1:** 碳青霉烯酶基因的引物序列及产物长度

基因引物	引物序列（5′→3′）	产物长度（bp）
*bla*KPC-F	CGTCTAGTTCTGCTGTCTTG	798
*bla*KPC-R	CTTGTCATCCTTGTTAGGCG	798
*bla*IMP-F	GGAATAGAGTGGCTTAAYTCTC	232
*bla*IMP-R	GGTTTAAYAAAACAACCACC	232
*bla*VIM-F	GATGGTGTTTGGTCGCATA	390
*bla*VIM-R	CGAATGCGCAGCACCAG	390
*bla*OXA-48-F	GCGTGGTTAAGGATGAACAC	438
*bla*OXA-48-R	CATCAAGTTCAACCCAACCG	438
*bla*NDM-F	GGTTTGGCGATCTGGTTTTC	621
*bla*NDM-R	CGGAATGGCTCATCACGATC	621
*bla*OXA-23-F	GATGTGTCATAGTATTCGTCG	606
*bla*OXA-23-R	TCACAACAACTAAAAGCACTG	606

**注** KPC：肺炎克雷伯菌碳青霉烯酶；IMP：亚胺培南酶；VIM：维罗纳整合子编码的金属-β-内酰胺酶；OXA：苯唑西林酶；NDM：新德里金属-β-内酰胺酶

6. 统计学处理：数据分析使用WHONET5.6软件。统计学分析采用R 4.4.1对不同科室单个菌种检出情况进行卡方检验或Fisher精确概率法，*P*<0.05为差异有统计学意义。

## 结果

一、主要病原菌分布

2020–2024年广东省56家医疗单位，血液内科、呼吸内科和ICU血液标本分离菌分别为8 968、6 440和25 511株。血液内科分离革兰阴性菌和革兰阳性菌分别占68.0％（6 095/8 968）、32.0％（2 873/8 968）。革兰阴性菌中肠杆菌目细菌占71.4％（4 353/6 095），主要以大肠埃希菌49.6％（2 160/4 353）和肺炎克雷伯菌36.0％（1 569/4 353）为主，非发酵菌以铜绿假单胞菌68.4％（1 052/1 539）、嗜麦芽窄食单胞菌13.7％（211/1 539）和鲍曼不动杆菌8.0％（124/1 539）为主。革兰阳性菌中检出率最高前三位分别为凝固酶阴性葡萄球菌47.4％（1 363/2 873）、金黄色葡萄球菌15.9％（457/2 873）和屎肠球菌5.6％（160/2 873）。见表2。5年间，主要革兰阴性菌和革兰阳性菌检出率基本保持稳定，凝固酶阴性葡萄球菌从2020年11.7％逐年上升至2024年17.7％（图1）。

**表2:** 2020–2024年广东省56家医院不同科室血液标本分离菌的构成

菌种	血液内科（8 968株）		呼吸内科（6 440株）		ICU（25 511株）		*P*值
	株数	占比（％）	株数	占比（％）	株数	占比（％）	
革兰阴性菌	6 095	68.0	2 876	44.7	9 931	38.9	<0.001
大肠埃希菌	2 160	24.1	1 520	23.6	3 504	13.7	<0.001
肺炎克雷伯菌	1 569	17.5	673	10.5	2 703	10.6	<0.001
铜绿假单胞菌	1 052	11.7	110	1.7	673	2.6	<0.001
嗜麦芽窄食单胞菌	211	2.4	18	0.3	263	1.0	<0.001
阴沟肠杆菌	163	1.8	39	0.6	260	1.0	<0.001
鲍曼不动杆菌	124	1.4	90	1.4	757	3.0	<0.001
其他	816	13.4	417	14.5	1 771	6.9	<0.001
革兰阳性菌	2 873	32.0	3 564	55.3	15 580	61.1	<0.001
葡萄球菌属	1 828	20.4	2 737	42.5	11 602	45.5	<0.001
凝固酶阴性葡萄球菌	1 363	15.2	2 218	34.4	9 114	35.7	<0.001
金黄色葡萄球菌	457	5.1	514	8.0	1 481	5.8	<0.001
链球菌属	570	6.4	457	7.1	1 258	4.9	<0.001
肺炎链球菌	60	0.7	95	1.5	162	0.6	<0.001
草绿色链球菌	30	0.3	20	0.3	63	0.2	<0.001
肠球菌属	259	2.9	225	3.5	2 147	8.4	<0.001
屎肠球菌	160	1.8	122	1.9	1 305	5.1	<0.001
粪肠球菌	70	0.8	85	1.3	716	2.8	<0.001
其他	224	2.5	150	2.3	1 580	6.2	<0.001

**注** ICU：重症监护病房

**图1:**
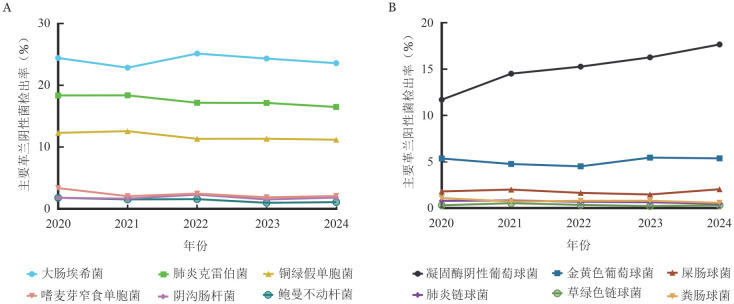
2020–2024年血液内科血液标本常见菌检出情况 **A** 主要革兰阴性菌检出率；**B** 主要革兰阳性菌检出率

与呼吸内科和ICU相比，血液内科血液标本分离的病原菌分布差异有统计学意义（均*P*<0.001）。血液内科革兰阴性菌检出率明显高于呼吸内科和ICU，尤其是肺炎克雷伯菌和铜绿假单胞菌检出率高，而凝固酶阴性葡萄球菌检出率显著低于呼吸内科和ICU。ICU中的屎肠球菌检出率明显高于血液内科和呼吸内科。

二、血液内科主要革兰阴性菌耐药性

1. 肠杆菌属细菌：5年监测中，大肠埃希菌对碳青霉烯类药物耐药率呈现上升的趋势，尤其美罗培南耐药率从2020年6.7％上升至2024年14.0％。第二、第三代头孢菌素类药物（除头孢他啶）和氟喹诺酮类药物耐药率高达49.7％，其中头孢西丁耐药率低于16.9％。大肠埃希菌对于加酶抑制剂复合药物耐药率<17.6％，对替加环素耐药率为0。肺炎克雷伯菌对碳青霉烯类药物耐药率也呈现上升趋势，尤其美罗培南耐药率从2020年5.8％上升至2024年15.8％。对大部分头孢菌素类药物的5年间耐药率保持稳定或略微下降（<35.1％）。对酶抑制剂复合药物耐药率升高，阿莫西林/克拉维酸和哌拉西林/他唑巴坦耐药率分别从2020年13.8％、12.7％上升至2024年18.5％和14.9％（表3）。

**表3:** 2020–2024年血液标本大肠埃希菌（ECO）和肺炎克雷伯菌（KPN）耐药率变迁

抗菌药物	2020年		2021年		2022年		2023年		2024年	
	ECO（*n*=407）	KPN（*n*=306）	ECO（*n*=389）	KPN（*n*=313）	ECO（*n*=446）	KPN（*n*=305）	ECO（*n*=465）	KPN（*n*=328）	ECO（*n*=453）	KPN（*n*=317）
阿米卡星	3.4	2.9	2.8	4.8	1.3	4.6	1.7	5.8	2.4	4.8
美罗培南	6.7	5.8	8.1	10.7	8.0	9.5	14.1	15.2	14.0	15.8
亚胺培南	4.9	3.9	5.4	7.1	5.4	7.3	8.5	10.1	7.6	8.6
厄他培南	3.9	2.3	5.1	5.7	5.0	6.6	8.5	9.3	6.4	6.9
头孢吡肟	30.5	17.0	26.7	25.9	29.4	21.7	27.5	23.5	30.7	21.5
头孢呋辛	56.9	30.1	49.0	33.5	55.1	29.3	49.7	35.1	52.2	30.9
头孢曲松	57.0	28.6	51.1	32.1	54.8	28.9	51.1	30.0	50.9	27.3
头孢他啶	24.3	17.5	24.7	21.9	23.1	18.9	24.0	23.9	21.9	20.2
头孢西丁	15.0	10.5	12.8	12.8	11.6	13.1	16.9	18.7	13.8	14.0
复方新诺明	66.3	43.4	69.0	47.2	71.8	46.2	68.3	44.0	65.6	42.0
替加环素	0.0	2.2	0.0	3.9	0.0	2.5	0.0	4.1	0.0	1.7
左旋氧氟沙星	50.2	21.5	53.5	28.3	55.3	23.6	51.5	28.0	50.3	24.0
阿莫西林/克拉维酸	14.3	13.8	13.8	16.9	11.8	15.8	17.6	17.3	14.2	18.5
哌拉西林/他唑巴坦	8.5	12.7	10.1	14.4	7.8	15.8	11.7	17.2	10.2	14.9
头孢哌酮/舒巴坦	9.7	10.1	9.4	14.8	9.6	12.9	13.3	15.1	9.0	11.5

2. 非发酵细菌：铜绿假单胞菌对常用抗菌药物整体耐药率呈现下降趋势，尤其美罗培南和亚胺培南耐药率从2020年的6.2％、10.2％下降至2024年的1.9％、6.1％；哌拉西林/他唑巴坦耐药率从11.0％下降至3.9％，其他大部分抗菌药物耐药率<10.0％。5年间，鲍曼不动杆菌对常见抗菌药物耐药变迁呈现先下降再上升的趋势，其中碳青霉烯类药物耐药率明显高于肠杆菌和铜绿假单胞菌；对头孢类和喹诺酮类药物耐药率从2020年20.0％和23.3％上升至2024年47.6％和42.1％。而对四环素类药物替加环素和米诺环素耐药率低，最高分别为5.6％和16.7％。见表4。嗜麦芽窄食单胞菌对常用抗菌药物耐药率呈现先上升再下降的趋势，左氧氟沙星和复方新诺明耐药率从2020年5.5％、1.9％上升至2021年11.4％、6.2％，再下降至2024年0。

**表4:** 2020–2024年血液标本铜绿假单胞菌（PAE）和鲍曼不动杆菌（ABA）耐药率变迁

抗菌药物	2020年		2021年		2022年		2023年		2024年	
	PAE（*n*=205）	ABA（*n*=30）	PAE（*n*=214）	ABA（*n*=26）	PAE（*n*=201）	ABA（*n*=28）	PAE（*n*=217）	ABA（*n*=19）	PAE（*n*=215）	ABA（*n*=21）
阿米卡星	0.5	NA	0.5	NA	0.5	NA	1.4	NA	0.5	NA
美罗培南	6.2	30.4	5.2	9.5	9.6	39.1	6.2	29.4	1.9	35.0
亚胺培南	10.2	30.0	9.8	19.2	10.0	37.0	8.8	27.8	6.1	38.1
头孢吡肟	2.9	20.0	0.9	19.2	4.0	39.3	1.4	21.1	1.4	42.9
头孢他啶	2.9	26.7	3.8	19.2	6.5	39.3	6.5	26.3	5.6	47.6
环丙沙星	4.5	26.9	2.8	13.0	4.5	37.5	4.7	29.4	2.4	42.1
左旋氧氟沙星	5.4	23.3	5.2	15.4	10.0	28.6	9.8	26.3	4.9	38.1
妥布霉素	0.6	27.3	0.6	17.6	1.8	29.4	1.7	15.4	0.5	15.4
复方新诺明	NA	40.0	NA	19.2	NA	39.3	NA	21.1	NA	42.9
替加环素	NA	0.0	NA	4.8	NA	4.5	NA	0.0	NA	5.6
米诺环素	NA	16.7	NA	0.0	NA	0.0	NA	0.0	NA	5.9
多黏菌素B	0.0	NA	0.0	NA	0.0	NA	5.3	NA	0.0	NA
哌拉西林/他唑巴坦	11.0	40	5.8	27.3	10.3	37.0	5.0	29.4	3.9	38.1
头孢哌酮/舒巴坦	4.3	25	4.4	9.5	7.3	39.1	5.0	14.3	4.1	27.8

**注** NA：不适用

三、血液内科主要革兰阳性菌耐药性

1. 葡萄球菌属：金黄色葡萄球菌对常见抗菌药物敏感率整体比凝固酶阴性葡萄球菌高，尤其金黄色葡萄球菌对喹诺酮类药物、庆大霉素、复方新诺明和利福平的耐药率<14.3％。两者均未发现万古霉素和替加环素的耐药株。见表5。

**表5:** 2020–2024年血液标本金黄色葡萄球菌（SAU）和凝固酶阴性葡萄球菌（CNS）耐药率

抗菌药物	2020年		2021年		2022年		2023年		2024年	
	SAU（*n*=89）	CNS（*n*=195）	SAU（*n*=81）	CNS（*n*=247）	SAU（*n*=80）	CNS（*n*=271）	SAU（*n*=104）	CNS（*n*=311）	SAU（*n*=103）	CNS（*n*=339）
青霉素G	85.2	91.4	87.3	90.6	81.6	91.6	93.0	91.3	88.1	91.1
红霉素	34.8	75.4	30.9	74.5	32.5	71.2	31.7	72.5	30.1	76.6
克林霉素	19.8	27.7	14.9	26.6	11.7	20.9	17.6	22.8	19.8	23.8
左氧氟沙星	6.0	42.1	10.0	41.7	6.9	44.6	12.1	40.1	10.8	49.3
环丙沙星	6.7	54.0	14.3	55.7	0.0	47.8	13.2	51.5	9.1	57.9
庆大霉素	3.4	12.5	7.4	14.2	3.8	11.9	5.8	15.5	6.8	16.0
复方新诺明	5.6	37.4	6.3	39.6	3.8	39.9	6.7	31.4	5.8	29.2
利福平	4.8	8.6	4.0	7.2	5.3	8.9	1.0	8.6	1.9	8.4
替考拉宁	0.0	0.0	0.0	0.0	0.0	0.5	0.0	0.0	0.0	0.7
利奈唑胺	0.0	0.0	0.0	0.0	0.0	0.4	0.0	0.0	0.0	0.0
万古霉素	0.0	0.0	0.0	0.0	0.0	0.0	0.0	0.0	0.0	0.0
替加环素	0.0	0.0	0.0	0.0	0.0	0.0	0.0	0.0	0.0	0.0

2. 肠球菌属：屎肠球菌对常见抗菌药物的整体耐药率比粪肠球菌高，其中屎肠球菌对万古霉素和替考拉宁的耐药率呈现上升的趋势，分别从2020年0和4.5％上升至2024年23.1％和21.9％。而粪肠球菌对万古霉素和替考拉宁近4年耐药率均为0。见表6。

**表6:** 2020–2024年血液标本屎肠球菌（EFM）和粪肠球菌（EFA）耐药率

抗菌药物	2020年		2021年		2022年		2023年		2024年	
	EFM（*n*=30）	EFA（*n*=18）	EFM（*n*=34）	EFA（*n*=12）	EFM（*n*=29）	EFA（*n*=14）	EFM（*n*=28）	EFA（*n*=15）	EFM（*n*=39）	EFA（*n*=11）
青霉素G	96.2	0.0	100.0	8.3	92.0	7.1	96.2	0.0	92.3	0.0
红霉素	78.6	50.0	85.3	58.3	78.6	42.9	78.6	53.3	82.1	40.0
环丙沙星	100.0	33.3	100.0	28.6	90.9	33.3	100.0	80.0	88.9	33.3
左旋氧氟沙星	95.5	25.0	100.0	40.0	95.2	44.4	93.8	37.5	91.7	28.6
高浓度庆大霉素	48.1	35.3	25.0	16.7	57.7	61.5	59.3	28.6	69.4	40.0
高浓度链霉素	37.5	25.0	45.5	0.0	50.0	0.0	33.3	20.0	25.0	100.0
呋喃妥因	50.0	11.1	54.5	0.0	50.0	NA	0.0	0.0	50.0	0.0
替考拉宁	4.5	10.0	0.0	0.0	20.0	0.0	20.8	0.0	21.9	0.0
万古霉素	0.0	5.6	0.0	0.0	17.2	0.0	17.9	0.0	23.1	0.0
利奈唑胺	0.0	7.7	3.3	9.1	0.0	0.0	3.7	6.7	0.0	10.0
替加环素	0.0	0.0	0.0	0.0	0.0	0.0	0.0	0.0	0.0	0.0

四、科室间CRO检出率比较

5年间，血液内科、呼吸内科和ICU中碳青霉烯耐药大肠埃希菌（CRECO）和碳青霉烯耐药肺炎克雷伯菌（CRKPN）的检出率呈上升趋势，其中血液内科CRECO检出率比呼吸内科和ICU高，碳青霉烯耐药铜绿假单胞菌（CRPAE）检出率在血液内科从2020年11.7％下降至2024年6.1％，呼吸内科出现先上升后下降的趋势，从2020年10.0％上升至2022年16.0％后下降至2024年9.5％，而ICU检出率维持稳定。血液内科中碳青霉烯耐药鲍曼不动杆菌（CRABA）从2020年30.0％上升至38.1％，而呼吸内科和ICU都呈现下降趋势。见图2。

**图2:**
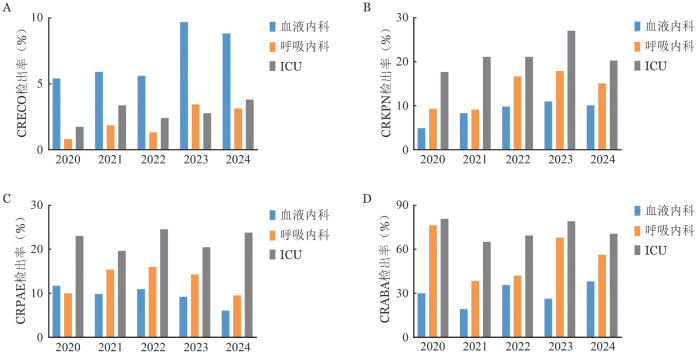
2020–2024年广东省56家医院血液标本不同科室碳青霉烯类耐药革兰阴性菌检出率情况 **A** CRECO；**B** CRKPN；**C** CRPAE；**D** CRABA **注** CRECO：碳青霉烯耐药大肠埃希菌；CRKPN：碳青霉烯耐药肺炎克雷伯菌；CRPAE：碳青霉烯耐药铜绿假单胞菌；CRABA：碳青霉烯耐药鲍曼不动杆菌；ICU：重症监护病房

五、主动监测CRO菌株耐药基因型和对新型抗菌药物敏感性分析

1. 主动监测CRO菌株耐药基因型：分层抽取血液标本来源CRO菌80株，其中CRECO、CRKPN、CRPAE、CRABA各20株。PCR检测碳青霉烯酶基因型发现，CRECO和CRPAE菌株分别携带B类金属酶*bla*NDM 80％和*bla*VIM 10％；CRKPN以A类丝氨酸基因型*bla*KPC为主，携带率为90％；CRABA菌株*bla*OXA-23携带率为100％。见图3。

**图3:**
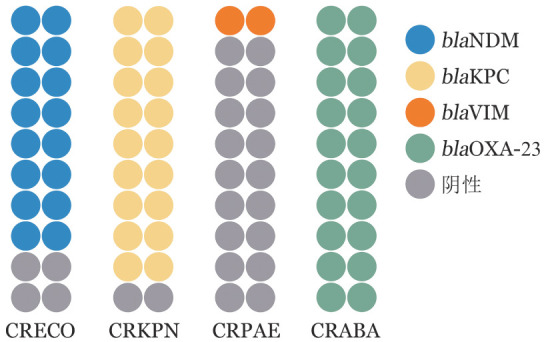
不同碳青霉烯类耐药革兰阴性杆菌（CRO）携带碳青霉烯酶基因分布情况 **注** CRECO：碳青霉烯耐药大肠埃希菌；CRKPN：碳青霉烯耐药肺炎克雷伯菌；CRPAE：碳青霉烯耐药铜绿假单胞菌；CRABA：碳青霉烯耐药鲍曼不动杆菌；NDM：新德里金属-β-内酰胺酶；KPC：肺炎克雷伯菌碳青霉烯酶；VIM：维罗纳整合子编码的金属-β-内酰胺酶；OXA：苯唑西林酶

2. 肠杆菌对新型抗菌药物敏感性分析：对CRO菌株进行体外药敏试验显示，CRECO对替加环素和多黏菌素的敏感率为100.0％，对头孢他啶/阿维巴坦50％最低抑菌浓度（MIC50）和90％最低抑菌浓度（MIC90）均>128 µg/ml，耐药率高达95.0％，对氨曲南/阿维巴坦MIC50和MIC90均≤1 µg/ml，敏感率达95.0％，舒巴坦/杜洛巴坦对产NDM CRECO的MIC50和MIC90均≤0.125 µg/ml。CRKPN对替加环素和多黏菌素敏感率分别为65.0％和100.0％。依拉环素对CRKPN的MIC50和MIC90均为1 µg/ml。在联合使用酶抑制剂时，发现对CRKPN的抑菌效果强于CRECO，氨曲南、亚胺培南和头孢他啶耐药率高达100.0％，而联合酶抑制剂氨曲南/阿维巴坦、亚胺培南/瑞莱巴坦和头孢他啶/阿维巴坦耐药率为0（表7）。

**表7:** 血液标本CRECO和CRKPN对抗菌药物的敏感性

抗菌药物	CRECO（*n*=20）				CRKPN（*n*=20）			
	MIC50（µg/ml）	MIC90（µg/ml）	耐药率（％）	敏感率（％）	MIC50（µg/ml）	MIC90（µg/ml）	耐药率（％）	敏感率（％）
庆大霉素	8	>64	55.0	20.0	>64	>64	75.0	25.0
阿米卡星	≤0.5	>64	35.0	65.0	>64	>64	75.0	25.0
氨曲南	4	>64	40.0	60.0	>64	>64	100.0	0.0
美罗培南	16	>64	100.0	0.0	>64	>64	100.0	0.0
亚胺培南	16	64	100.0	0.0	64	>64	100.0	0.0
替加环素	≤0.5	≤0.5	0.0	100.0	2	4	0.0	65.0
依拉环素	NA	NA	NA	NA	1	1	0.0	100.0
多黏菌素B	1	2	0.0	100.0	1	2	0.0	100.0
头孢他啶/阿维巴坦	>128	>128	95.0	5.0	4	8	0.0	100.0
氨曲南/阿维巴坦	≤1	≤1	0.0	95.0	≤1	2	0.0	100.0
亚胺培南/瑞莱巴坦	8	32	100.0	0.0	≤0.5	1	0.0	100.0
头孢哌酮/舒巴坦	>128	>128	100.0	0.0	>128	>128	100.0	0.0
舒巴坦/杜洛巴坦	≤0.125	≤0.125	NA	NA	NA	NA	NA	NA

**注** CRECO：碳青霉烯类耐药大肠埃希菌；CRKPN：碳青霉烯类耐药肺炎克雷伯菌；MIC：最低抑菌浓度；NA：不适用

3. 碳青霉烯类耐药非发酵菌：CRPAE对阿米卡星和多黏菌素B敏感率高达95.0％，氨曲南MIC50和MIC90分别为32 µg/ml和64 µg/ml，而氨曲南/阿维巴坦MIC50和MIC90分别为16 µg/ml和32 µg/ml，抑菌效果是氨曲南的两倍；亚胺培南耐药率为95.0％，而亚胺培南/瑞莱巴坦耐药率直接下降至10.0％；头孢他定/阿维巴坦对CRPAE的抑菌效果不明显。CRABA对美罗培南、亚胺培南、左氧氟沙星和头孢哌酮/舒巴坦耐药率高达100.0％，而舒巴坦/杜洛巴坦敏感率高达100.0％。其中依拉环素MIC50和MIC90分别为1 µg/ml和2 µg/ml，抑菌效果是替加环素的4倍（表8）。

**表8:** 血液标本碳青霉烯类耐药铜绿假单胞菌和鲍曼不动杆菌对抗菌药物的敏感性

抗菌药物	CRPAE（*n*=20）				CRABA（*n*=20）			
	MIC50（µg/ml）	MIC90（µg/ml）	耐药率（％）	敏感率（％）	MIC50（µg/ml）	MIC90（µg/ml）	耐药率（％）	敏感率（％）
氨曲南	32	64	65.0	10.0	NA	NA	NA	NA
美罗培南	16	32	80.0	0.0	64	128	100.0	0.0
亚胺培南	16	>128	95.0	5.0	64	64	100.0	0.0
阿米卡星	2	8	0.0	100.0	NA	NA	NA	NA
头孢他啶	8	>128	45.0	50.0	NA	NA	NA	NA
替加环素	NA	NA	NA	NA	4	8	20.0	35.0
依拉环素	NA	NA	NA	NA	1	2	15.0	85.0
米诺环素	NA	NA	NA	NA	16	32	60.0	0.0
多黏菌素B	2	2	5.0	95.0	0.5	1	0.0	NA
氯霉素	>64	>64	NA	NA	NA	NA	NA	NA
左氧氟沙星	2	32	50.0	20.0	16	64	100.0	0.0
头孢他啶/阿维巴坦	8	128	45.0	55.0	NA	NA	NA	NA
氨曲南/阿维巴坦	16	32	25.0	40.0	NA	NA	NA	NA
亚胺培南/瑞莱巴坦	≤0.5	4	10.0	85.0	NA	NA	NA	NA
头孢哌酮/舒巴坦	16	64	40.0	50.0	128	128	100.0	0.0
舒巴坦/杜乐巴坦	NA	NA	NA	NA	1	2	0.0	100.0

**注** CRPAE：碳青霉烯耐药铜绿假单胞菌；CRABA：碳青霉烯耐药鲍曼不动杆菌；MIC50：50％最低抑菌浓度；MIC90：90％最低抑菌浓度；NA：不适用

## 讨论

2020–2024广东省56家医院血液内科血液标本分离菌研究结果显示，革兰阴性菌占68.0％，与国内外研究报道一致，革兰阴性菌仍处于血液内科血流感染的主导地位。大肠埃希菌是最常见的病原体、其次肺炎克雷伯菌和铜绿假单胞菌。革兰阳性菌以凝固酶阴性葡萄球菌、金黄色葡萄球菌和屎肠球菌为主[10]。5年间，主要革兰阴性菌和革兰阳性菌检出率基本维持稳定，但凝固酶阴性葡萄球菌检出率呈现逐年上升趋势。由于血液病患者常需接受导管植入、骨髓穿刺等侵入性操作，其凝固酶阴性葡萄球菌的定植或污染风险增加，可能导致临床误判为感染，错误使用抗生素[11]。研究数据显示，2021年1月开始广东省临床分离的屎肠球菌对万古霉素的耐药率呈现显著上升趋势，达到20％～50％[12]，这一发现与本研究结果具有一致性。万古霉素耐药的屎肠球菌快速增长，可能是屎肠球菌在医院环境中有极强的适应性，可通过环境污染、无症状定植和临床感染等多种形式持续存在，而且在进化过程中可以形成具有选择优势的克隆株，这些克隆株不仅适应能力更强，还具备广泛的传播潜力[13]–[14]。

肠杆菌感染是血液内科血流感染的主要病原体，而肠杆菌属细菌对碳青霉烯类药物耐药率呈现升高趋势，这对血液科抗感染极具挑战。本研究发现，CRECO和CRKPN的检出率从2020年至2024年都明显上升，高于国内前期流行病学数据[15]–[16]。血液病患者长期住院、免疫力低下和造血功能障碍等，会增加碳青霉烯类耐药菌感染的发生率，尤其合并血流感染容易造成急性休克、弥漫性血管内凝血和多功能障碍，死亡率高达65％[17]。

本研究采用分层抽样检测了CRO的碳青霉烯酶分子表型，其结果与国内外分子流行病学结果一致：CRECO以产NDM为主，CRKPN以产KPC为主[18]。针对CRO新型抗菌药的体外药敏结果显示，本研究抽样菌株对新抗菌药都保持很高敏感性。氨曲南/阿维巴坦对产金属酶的CRO抗菌活性强。超过85％的产金属-β-内酰胺酶分离株对氨曲南/阿维巴坦敏感，而单独使用氨曲南的敏感率仅有6％[19]–[20]。本研究发现CRECO对头孢他啶/阿维巴坦耐药率高达95.0％，而对氨曲南/阿维巴坦敏感率高达95.0％。本研究发现产NDM的CRECO对舒巴坦/杜洛巴坦MIC50和MIC90均≤0.125 µg/ml，与国外研究一致[21]。舒巴坦/杜洛巴坦适应证为CRABA感染，其对携带NDM的CRECO有较强的体外抗菌活性机制应进一步研究。

本研究因诸多原因，存在一定局限性。首先，针对CRO的新型抗菌药物的菌株样本量较少，未必能全面反映CRO对新药敏感性，未来有必要扩大菌株量，了解新型抗菌药物治疗血液科血流感染的潜力。此外，因KPC变异体逐渐增多，可能对新型酶抑制剂耐药[22]。而现有KPC快检方法无法检出KPC各种变异体，因此分子检测结合微生物培养和药敏，对临床精准治疗更重要。

综上，血液内科血液标本病原菌分布广泛，且部分抗菌药物耐药率逐年升高，尤其CRO检出率呈升高趋势，但对抗CRO的新型抗菌药保持高度敏感。因此要加强对血液标本分离菌的耐药监测，合理应用抗菌药物，提高经验性治疗的疗效，并强化医院感染控制措施，降低患者感染耐药菌的发生率和死亡率。
